# BMX: a tool for computing bacterial phyletic composition from orthologous maps

**DOI:** 10.1186/s13104-015-1017-z

**Published:** 2015-02-24

**Authors:** Benard W Kulohoma

**Affiliations:** Institute of Infection and Global Health, Liverpool, University of Liverpool, Liverpool, 8 West Derby Street, Liverpool, L69 7BE UK; International Centre for Insect Physiology and Ecology, P.O. Box 30772–00100, Nairobi, Kenya; Centre for Bioinformatics and Biotechnology, University of Nairobi, P.O. Box 30197–00100, Nairobi, Kenya

**Keywords:** Orthologous maps, Core genome, Prokaryotes

## Abstract

**Background:**

New sequencing technologies have made it possible to explore genetic diversity at higher resolution in microbial populations. However, our understanding evolutionary relationships, and comparison of closely and distantly related bacterial genomes from these massive datasets remains a formidable challenge. Numerous clustering algorithms that group genomic data based on homology have been developed, but new tools are still required to analyse the resultant orthologous maps to understand functional genetic similarities and their phyletic patterns (patterns of presence of absence of genes).

**Findings:**

Bacterial Makeup eXplorer (BMX) implements an algorithm that swiftly and efficiently facilitates the determination of the number of orthologs in prokaryotic genomes employing a reference free approach, which may be further exploited to transfer of gene annotations. BMX is able to integrate orthologous maps of highly diverse prokaryotic genomes therefore making it possible to perform robust and scalable, multi-platform, high quality annotation transfer and gene-by-gene composition assessment method. In addition results are presented in the form of publication quality figures.

**Conclusions:**

BMX allows extensive data analysis of orthologous map databases to understand underlying biological relationships. Furthermore, BMX is portable across different platforms and can be installed easily. In summary, BMX allows higher resolution analysis of genomes from diverse bacterial populations

**Electronic supplementary material:**

The online version of this article (doi:10.1186/s13104-015-1017-z) contains supplementary material, which is available to authorized users.

## Findings

### Background

The concept of orthology provides an important basis for studying mechanisms of bacterial genome evolution, functional genetics, and biological networks. Orthologs are genes descended from a common ancestor as a result of speciation, which are likely to retain the same function in the course of evolution [1,2]. They contrast with paralogs, which may retain the same function or evolve new functions that are related to or different from the original function due to lack of the original selective pressure on one or more copies of the duplicated genes [1-3]. However, it is important to note that function retention among orthologs has some notable exceptions [4]. Determination of orthology between bacterial genomes provides a detailed understanding of bacterial population genetics and evolutionary biology [5,6]. This has greatly been enhanced by recent rapid and cost-effective advances in sequencing technologies, which have provided highly accurate and reproducible large-scale datasets required to unravel the broad spectrum of genetic diversity from different microbes [7]. These datasets have allowed higher resolution analysis of large bacterial populations, and significantly improved our understanding of diversity between and across species.

Numerous computational methods that accurately and efficiently infer orthology from sequenced genomes have been developed [8,9]. However, effective algorithms are still required to gain functional insight from the extensive generated homologous maps [10]. Recently, more emphasis has been redirected towards identification of orthologs, which are crucial elements of comparative genomics applications [6,11,12]. Ortholog annotation allows easy comparison of genomes and can highlight key conserved antigens and virulence complements found in the genomes of clinically important bacterial strains under study, which could be exploited as novel potential targets for therapeutic approaches [13-15].

We describe Bacterial Makeup eXplorer (BMX), a highly efficient, stand alone tool that can be used to easily and effectively compute the size and composition of the genes common to all strain genomes, within each gene cluster under study (referred to henceforth as the core genome) from OrthoMCL [16] generated orthologous gene maps. Most genome alignment approaches require high quality reference sequences and are problematic when comparing distantly related species or species with high rates of recombination [6]. BMX is a robust reference-free highly scalable approach capable of integrating datasets containing highly divergent genomes. Moreover, BMX can also be exploited as an annotation transfer tool by including a known well-annotated reference genome in the dataset, thereby allowing inference of a gene cluster function. Its features include: data reorganization, genome clusters quality assessment, iterative random genome sampling, and core and accessory genome distinction, and size and composition computation; generating accurate molecular barcodes from whole genome sequence data.

### Implementation

**Figure 1:**
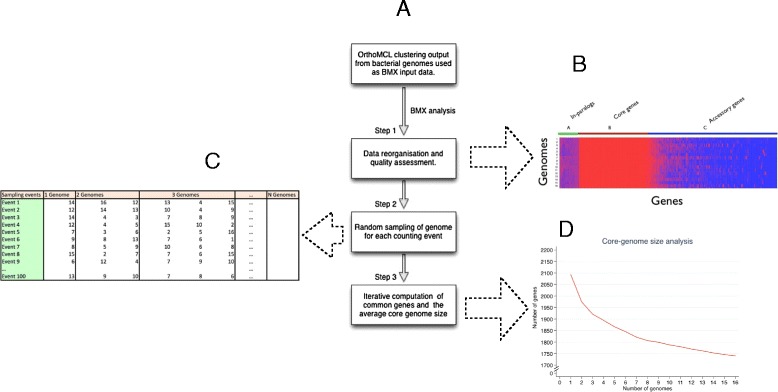
**A flow-chart describing the pipeline implemented in BMX. (A)** The pipeline consists of three mains steps that involve (i) reorganisation and quality assessment of the data, (ii) random sampling of genomes for each computation event, and (iii) iterative computation of the core and accessory genome size and composition. These are marked as step 1 through 3 respectively. **(B)** A quality assessment heatmap is generated at step 1, with genes represented in red and the absence of genes represented in blue. The core genome is represented by solid red columns of genes. Corrupted or poor quality genomes can be visually identified, for instance as having missing genes in the core genomes. **(C)** During the core genome analysis, genomes in the dataset are selected at random from the dataset and each genome (represented by a number) is selected only once. The calculation of the core genome size of N genomes is performed N times, starting with determination of the size determination of one genome up to N genomes, with an increment of 1 genome is each subsequent calculation. This is known as an event and is implemented as an arithmetic progression. 100 such events are performed and the average value is used to establish the core genome size of N genomes. **(D)** A graph of the core genome size is generated. The files showing the core genome sizes (“CG.txt”) and accessory clusters (“not_CG.txt”), which do not form part of the core genome, are also generated.

BMX is implemented in Perl, BioPerl and R, and includes a bash script wrapper. The user supplies an orthologous map input file of interest in OrthoMCL format for analysis, and the number of genomes under study. OrthoMCL format is used for convenience and uniformity, and orthologous maps in other database formats could be easily converted to this input format. However, updates to BMX are anticipated soon after the ortholog database community fully embraces and implements a standardised XML database format. Although various ortholog databases have signed-up for and agreed to use XML, there currently is still community efforts towards adoption of standard file formats and benchmarking [17]. Detailed step-by-step documentation that includes input files and how to run the software is also provided. Figure 1 portrays the entire BMX pipeline in pseudo-code. Ortholog clusters are first organized into a matrix of genome content, while taking into account absent genes. In this matrix the matrix columns represent different strain genomes, while the rows represent the orthologous clusters across the genomes. The composition of core (genes common to all strain genomes) and accessory (genes not common to all strain genomes) gene clusters is determined using randomly sampled genomes (the matrix columns) iteratively, in an arithmetic progression fashion using the mathematical formula:

$$ {S}_N = N/2\left[2{a}_1 + \left(N-1\right)\ d\right] $$

The number of random sampling events when determining the core genome size given *N* number of genomes, *S*_*N*_, is established using the least number of genomes under consideration, which is set to 1 genome by default, *a*_*1*_, the total number of genomes under consideration (i.e. the dataset size), *N*, and the common difference of successive genomes, *d*, (i.e. 1) used during sampling. During random sampling, the total number of genomes, *N*, is initialized as 1 genome for the first event, and increased by one unit for each of the subsequent events, until it is equivalent to the total number of genomes in the dataset in the final event. The random genomes are only sampled once during each event and the total number of orthologs shared by all genomes counted once for each cluster, thereby excluding paralogous counts. The default setting uses 100 iterations resulting in 100 different input orders per event, and the more advanced users may alter this value depending on their preference, in the BMX script L.pl. However, it’s important to note previous tests show that 100 iterations give the optimal results. The average core genome size per event is computed enabling correlation with the number of genomes sampled. Poor quality genomes can be identified for exclusion, by visual examination of a quality control diagram generated using R scripts. Core and accessory genome size and composition comparisons between different datasets can be achieved by this approach, thereby allowing annotations to be easily transferred to genes of draft genomes. A plot of the core genome size, which only consists of clusters that have a gene in every taxon, is generated using R scripts.

### Results and discussion

BMX has already been used on highly divergent bacterial genomes of *Streptococcus pneumoniae* (pneumococcus) (Additional file 1: Table S1). Pneumococci are clinically important and have highly recombigenic genomes from multiple diverse lineages, which make it challenging to study their evolutionary history [18]. BMX was used to assess the composition of 140 invasive disease pneumococcal genomes. Datasets of 16, 70 and 140 Streptococcus genome maps are included in the software download itself, and can be found at this url: http://sourceforge.net/projects/bmexplorer/files/latest/download. The estimated run times for analysis of these datasets using BMX are: 16 genomes in less than 3 mins, 70 genomes in 15–20 mins, and 140 genomes in 45 mins on a 2.7 GHz Intel Core i7 processor and 8Gb 1333 MHz DDR3 memory. Paralogs, core genes and other accessory genes were clearly distinguished, allowing the subsequent robust reconstruction of phylogenetic trees. BMX can be exploited to identify relevant functional sets of core genes, which encode important virulence complements always present in the genome that can be exploited as therapeutic and diagnostic targets.

### Conclusion

BMX is a scalable, reference-free framework for computing gene-by-gene core and accessory genome composition and making comparisons between datasets. We believe that BMX will facilitate hypothesis generation and design of new experiments that explore the genomic diversity of bacteria, and its use can be extended to other prokaryotic organisms.

## Availability and requirements

**Project name:** BMX.

**Project home page:**http://sourceforge.net/projects/bmexplorer/

**Operating system(s):** Multiple platforms.

**Programming language:** Perl, BioPERL and R.

**Other requirements:** The following modules must be installed in perl: Bio::Perl, IO::String, Bio::SeqIO, List::Util, List::Util ‘max’, Text::CSV, integer.

The following libraries/packages must be installed in R: RColorBrewer, gplots.

**License:** GNU GPL.

**Any restrictions to use by non-academics:** none.

**Availability of supporting data**

The data set of the orthologous map from 140 invasive disease pneumococcal genomes supporting the results of this article is available in the publically accessible source forge repository, http://sourceforge.net/projects/bmexplorer/.

## Additional file

Additional file 1: Table S1. The 213 publically available Streptococcus pneumoniae genomes used a test datasets for the BMX program. OrthoMCL was used to generate orthologous maps from these dataset.
